# A comprehensive analysis of different types of databases reveals that *CDH1* mRNA and E-cadherin protein are not downregulated in most carcinoma tissues and carcinoma cell lines

**DOI:** 10.1186/s12885-023-10916-0

**Published:** 2023-05-15

**Authors:** Brihget Sicairos, Shorna Alam, Yuchun Du

**Affiliations:** 1 Department of Biological Sciences, University of Arkansas, Fayetteville, AR 72701 USA; 2 Bentonville West High School, Centerton, AR 72719 USA; 3 Present address: Department of Electrical Engineering and Computer Science, Massachusetts Institute of Technology, Cambridge, MA 02139 USA

**Keywords:** *CDH1*, E-cadherin, Tumor suppressor, Carcinoma, Metastasis, Tumor progression, Gene expression

## Abstract

**Background:**

The *CDH1* gene codes for the epithelial-cadherin (E-cad) protein, which is embedded in the plasma membrane of epithelial cells to form adherens junctions. E-cad is known to be essential for maintaining the integrity of epithelial tissues, and the loss of E-cad has been widely considered a hallmark of metastatic cancers enabling carcinoma cells to acquire the ability to migrate and invade nearby tissues. However, this conclusion has come under scrutiny.

**Methods:**

To assess how *CDH1* and E-cad expression changes during cancer progression, we analyzed multiple large transcriptomics, proteomics, and immunohistochemistry datasets on clinical cancer samples and cancer cell lines to determine the *CDH1* mRNA and E-cad protein expression profiles in tumor and normal cells.

**Results:**

In contrast to the textbook knowledge of the loss of E-cad during tumor progression and metastasis, the levels of *CDH1* mRNA and E-cad protein are either upregulated or remain unchanged in most carcinoma cells compared to normal cells. In addition, the *CDH1* mRNA upregulation occurs in the early stages of tumor development and the levels remain elevated as tumors progress to later stages across most carcinoma types. Furthermore, E-cad protein levels are not downregulated in most metastatic tumor cells compared to primary tumor cells. The *CDH1* mRNA and E-cad protein levels are positively correlated, and the *CDH1* mRNA levels are positively correlated to cancer patient’s survival. We have discussed potential mechanisms underlying the observed expression changes in *CDH1* and E-cad during tumor progression.

**Conclusions:**

*CDH1* mRNA and E-cadherin protein are not downregulated in most tumor tissues and cell lines derived from commonly occurring carcinomas. The role of E-cad in tumor progression and metastasis may have previously been oversimplified. *CDH1* mRNA levels may serve as a reliable biomarker for the diagnosis of some tumors (such as colon and endometrial carcinomas) due to the marked upregulation of *CDH1* mRNA in the early stages of tumor development of these carcinomas.

**Supplementary Information:**

The online version contains supplementary material available at 10.1186/s12885-023-10916-0.

## Background

*CDH1* is a gene that codes for the epithelial-cadherin (E-cad) protein, which is embedded in the plasma membrane of epithelial cells forming the tissues that cover the body surfaces and line the walls of cavities, channels, and glands [1, 2]. E-cad is a calcium-dependent cell–cell adhesion protein that forms homophilic interactions in adjacent epithelial cells establishing adherens junctions [3, 4]. This protein plays a major role in embryonic development and morphogenesis [5, 6]. In its inactive form, E-cad contains a short signal sequence for import to the endoplasmic reticulum (ER), a 130 amino acid pro-peptide, a single transmembrane domain, a 150 amino acid cytoplasmic domain, and a 550 amino acid ectodomain [7, 8]. E-cad is activated after cleavage of the pro-peptide in the presence of calcium ions [9]. In the extracellular matrix (ECM), the ectodomains of E-cad on adjacent cells bind each other to form adherens junctions, while the cytoplasmic domain of E-cad interacts with β-catenin, which in turn binds α-catenin connecting to the actin cytoskeleton of the cell leading to the stabilization and integrity of the epithelial tissues [10, 11].

The loss of E-cad expression has been considered a hallmark of cancer progression and metastasis [12, 13] via loss of heterozygosity of the chromosomal region 16q22.1 containing the *CDH1* locus, nonsense mutations [14], or promoter methylation [15]. E-cad activity as a tumor suppressor manifests via its loss during epithelial-mesenchymal transition (EMT) and/or regulation during metastatic progression, where its loss leads to increased tumor cell migration and invasion [16]. E-cad also plays a role in primary tumor development, progression [17, 18], and metastatic colonization [19]. The loss of E-cad expression is thought to disrupt adherens junctions leading to the acquisition of motility/invasiveness of metastasizing tumor cells. Although some carcinoma cells undergo EMT, many carcinoma cells neither fully lose the ability to produce E-cad nor undergo a mesenchymal‐to‐epithelial transition (MET) during metastasis [20–22]. Assertions as to the necessity of EMT and its reverse MET in metastasis have been controversial [23–25] as many metastatic tumor cells still express E-cad [26–29].

In this study, we analyzed multiple large transcriptomics, proteomics, and immunohistochemistry datasets on clinical cancer samples and cancer cell lines to determine the levels of *CDH1* mRNA and E-cad protein in different carcinomas during tumor progression. Strikingly, the levels of *CDH1* mRNA and E-cad protein were not reduced in most of the examined tumors, even in the later stages of cancer compared to respective healthy tissues. The only exception to this trend was kidney cancer, which exhibited significantly lower levels of *CDH1* mRNA and E-cad protein, the pattern normally described in textbooks. The observations presented in this study demonstrate that the changes in E-cad expression during tumor progression and metastasis are more complex than widely believed.

## Methods

### Analysis of *CDH1* mRNA levels in cancer clinical samples and cancer cell lines

The Gene Expression Profiling Interactive Analysis 2 (GEPIA2) web server (http://gepia2.cancer-pku.cn/#index) [30, 31] was used to analyze levels of *CDH1* mRNA in the tumors of interest. GEPIA2 is a resource for gene expression analysis compiling tumor and normal samples from The Cancer Genome Atlas (TCGA), a database containing samples from 11,000 patients, and the Genome-Typing Expression (GTEx), a database containing 948 post-mortem donors and approximately 17,382 RNA sequencing (RNA-seq) samples across 54 tissue sites and 2 cell lines [32]. *CDH1* mRNA levels between tumor and normal samples for breast, colon, lung, ovarian, pancreatic, endometrial, kidney, liver, and head/neck tissues were analyzed using one-way analysis of variance (ANOVA). A change was considered significantly different if i) the log_2_ fold change was larger than 1 (representing an actual fold change of 2), ii) the *q*-value was smaller than 0.01 or the *p*-value was smaller than 0.05, and iii) the samples were available in both the TCGA and GTEx databases. The *CDH1* mRNA expression in tumor subtypes relative to corresponding normal tissues was analyzed with the same parameter as discussed above. *CDH1* mRNA expression across specific tumor stages was performed using a one-way ANOVA, in which expression was compared among the major stages of specific carcinomas. Only the major stages containing enough samples for statistical analysis were analyzed.

The Cancer Dependency Map (DepMap, https://depmap.org/portal/), which systematically identifies genetic and pharmacologic dependencies and biomarkers for 1,072 cancer cell lines from various lineages [33], was used to analyze the *CDH1* mRNA profile in cell lines derived from breast, colorectal, endometrial, head/neck, kidney, lung, liver, pancreatic, and ovarian carcinomas. The *CDH1* mRNA levels in the kidney, breast, liver, colorectal, ovarian, pancreatic, endometrial, and lung carcinomas cell lines were analyzed using the Cancer Cell Line Encyclopedia (CCLE) RNA-seq data available on the European Molecular Biology Laboratory’s European Bioinformatics Institute (EMBL-EBI) database (https://www.ebi.ac.uk/gxa/experiments/E-MTAB-2770/) with the expression value set at 0.5 and the data reported as transcripts per million (TPM) [34]. The results were reported in TPM where low/no expression was defined as 0–10 TPM, medium expression was defined as 11–100 TPM, and high expression was defined as 101–2,120 TPMs. The cell lines used are listed in Supplementary Tables—Additional file 2 (the same for other sections that were involved in using cell lines), and the sources of the cell lines were described in the respective studies.

### *CDH1* promoter methylation analysis in tissue samples

The University of Alabama at Birmingham Cancer Data Analysis Portal (UALCAN, http://ualcan.path.uab.edu/analysis-prot.html), an interactive web source for analyzing cancer OMICS data with a focus on transcriptomics and proteomics, and the TCGA methylation datasets (http://ualcan.path.uab.edu/analysis.html) were used to assess promoter methylation in kidney carcinomas compared to normal samples, and among the major stages of kidney carcinoma [35, 36]. The beta value, the ratio of methylated probe intensity to the total probe intensity (the sum of methylated and unmethylated probe intensity), was reported. The beta values of CpG probes ([TSS200, TSS1500]) located up to 1,500 bp upstream of the *CDH1* gene’s start site were plotted. Beta values range from 0 (unmethylated) to 1 (fully methylated).

### Analysis of E-cad protein levels in clinical cancer samples, cancer cell lines, and normal samples

The expression levels of E-cad protein in cancer and normal tissues were determined using the Clinical Proteomic Tumor Analysis Consortium (CPTAC) Confirmatory/Discovery dataset available on the UALCAN web-server (http://ualcan.path.uab.edu/analysis-prot.html) [37]. The log2 spectral count ratios obtained from CPTAC were normalized within each sample profile and then normalized across samples. The results were presented using *Z*-values, the standard deviation from the median across samples for any given carcinoma compared to normal tissues. Differential expression of E-cad with *p*-values smaller than 0.05 was considered significant.

The expression of E-cad in cancer cell lines was determined using the proteomics data available in the DepMap portal (https://depmap.org/portal/interactive/) produced from the quantitative analyses of protein expression in 375 cancer cell lines in the CCLE [38]. The protein expression is reported as values closely related to log2-transformed ratios to the bridge, a sample of 10 cell lines from the CCLE selected for maximal protein expression diversity to help with the normalization of protein levels in the cell lines analyzed [38]. A cell line was said to have a high expression of E-cad if the log2-transformed values were higher than 0, and a low expression if the log2-transformed values were lower than 0. In this context, 0 means that there is no difference between the expression of E-cad in the cell line of interest compared to the levels of E-cad in the bridge mixture.

To analyze whether the levels of E-cad were different between metastatic and primary carcinoma cell lines, the E-cad expression data was downloaded from the DepMap portal (Proteomics data) [38], then cancer cell lines derived from breast, colon, head/neck, lung, ovarian, and endometrial carcinomas were separated into primary tumor cell lines and metastatic cell lines. The mean E-cad expression for the defined groups in specific lineages was determined and analyzed using one-way ANOVA using the PSI-Plot software. Analyses for kidney and liver carcinoma cell lines were not performed because of the lack of enough metastatic cell lines to calculate statistical significance. Metastatic and primary cell lines from all lineages were compared also using one-way ANOVA; this comparison included metastatic and primary cell lines derived from kidney and liver cancer as well. *p*-value less than 0.05 represented significant differential expression between primary and metastatic carcinoma cell lines.

### Analysis of immunohistochemistry staining of E-cad in clinical cancer tissue samples

E-cad expression in immunohistochemically stained with CAB072856 antibody, and pathologist-certified/annotated images for breast, colon, head/neck, kidney, lung, liver, pancreatic, ovarian, and endometrial carcinomas, and corresponding normal tissue microarrays stored on the Human Protein Atlas (HPA) (https://www.proteinatlas.org/) was assessed [39]. The HPA summarized the results as high, medium, and low/not detected E-cad staining intensity.

### Determination of the relationship between *CDH1* mRNA levels and E-cad protein levels in cancer cell lines

Pearson Correlation Analysis and Spearman Correlation Analysis were carried out using the DepMap portal (https://depmap.org/portal/interactive/) to compare the *CDH1* mRNA and E-cad protein levels in 9 individual carcinoma cell lineages, including endometrial, head/neck, pancreatic, ovarian, liver, kidney, colorectal, breast, and lung tissues [33, 38]. The relationship between *CDH1* mRNA levels and E-cad protein levels in all the carcinoma cell lines was also determined.

### Determination of the relationship between *CDH1* mRNA levels in carcinoma tissues and cancer patient’s survival

GEPIA2 (http://gepia2.cancer-pku.cn/#index) was used to assess the relationship between *CDH1* mRNA levels in carcinoma tissues and patient’s overall survival (OS) and disease-free survival (DFS) [30, 31]. GEPIA2 employs the Log-rank test, also known as the Mantel-Cox test, to assess the null hypothesis that there is no significant difference in survival among the different groups being compared [30, 31]. Cancer patients with defined types of cancer were divided equally into two groups based on the median level of *CDH1* mRNA: the high *CDH1* mRNA group represented half of the patients with tumors expressing higher levels of *CDH1* mRNA, and the low *CDH1* mRNA group represented half of the patients with tumors expressing lower levels of *CDH1* mRNA relative to the median *CDH1* mRNA level in the group. A *p*-value less than 0.05 was considered significant, meaning that the null hypothesis was rejected and that there was a significant difference in the survival between the two groups.

### Statistical analysis

The difference in *CDH1* mRNA expression between carcinoma and normal tissues, between carcinoma subtypes and normal tissues, across major cancer stages, and between metastatic and primary cancer cell lines were analyzed with one-way ANOVA. Survival analyses were performed using a Log-rank test (Mantel-Cox test) [30, 31]. The difference in E-cad expression and promoter methylation between carcinoma types and normal tissues, and between stages and normal tissues were analyzed using a t-test [35–37, 40]. The RNA seq data and proteomic data on the sites have been normalized by the authors of the sites. The correlation between *CDH1* mRNA and E-cad protein expression was analyzed using both Pearson correlation analysis and Spearman correlation analysis [33, 38].

## Results

### *CDH1* mRNA is upregulated or unchanged in most carcinoma tissues

**Fig. 1:**
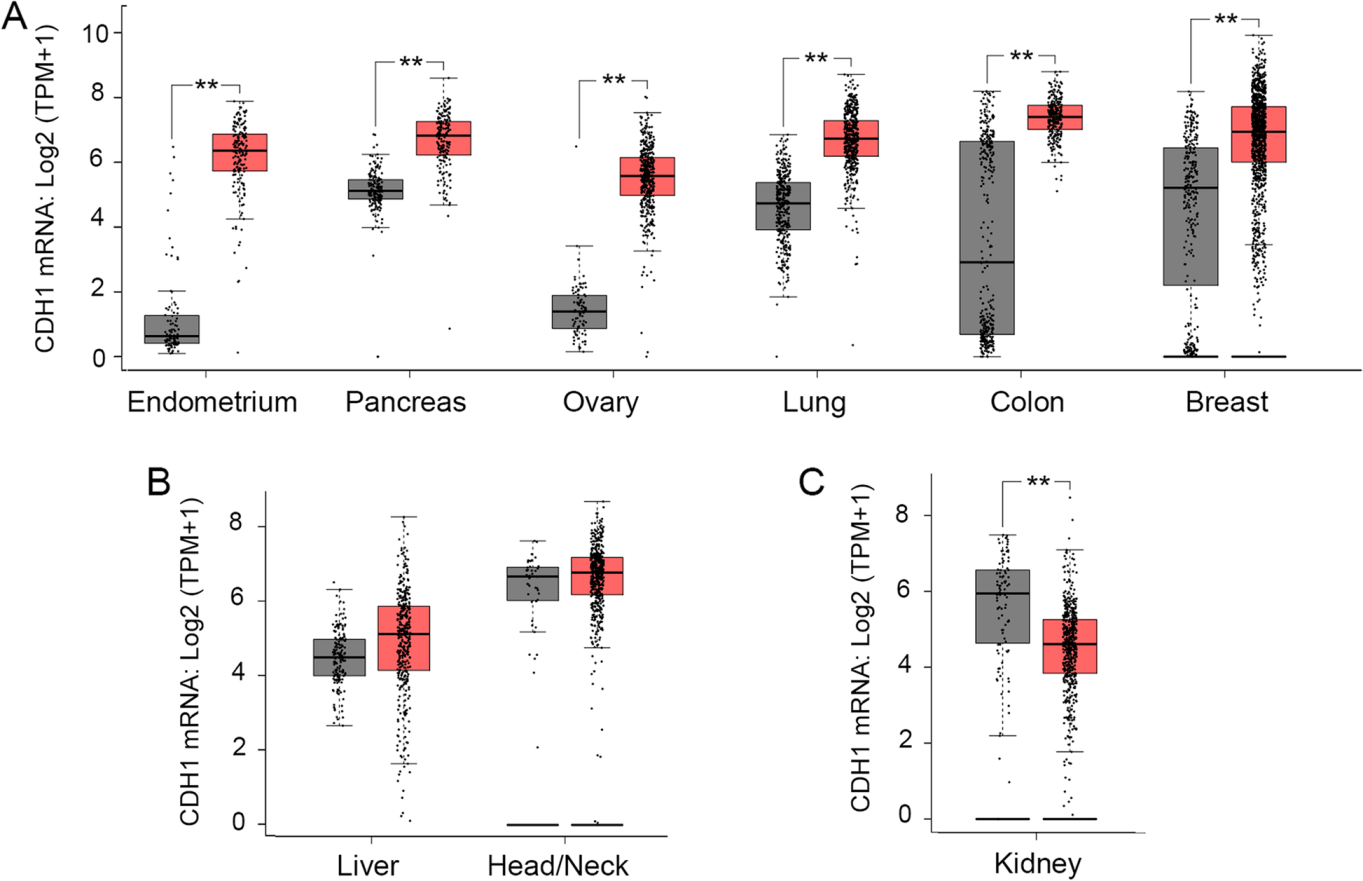
*CDH1* mRNA is either upregulated or remains unchanged in most carcinoma tissues. **A**, *CDH1* mRNA is significantly upregulated in tissues derived from endometrial (number of normal tissues, *N* = 91; number of tumor tissues, T = 174), pancreatic (*N* = 171; T = 179), ovarian (*N* = 88; T = 426), lung (*N* = 347; T = 483), colon (*N* = 349; T = 275), and breast (*N* = 281; T = 1,085) carcinomas compared to the corresponding normal tissues. **B**, *CDH1* mRNA remains unchanged in the liver (*N* = 160; T = 369) and head/neck (HNSC) (*N* = 44; T = 519) carcinomas compared to the corresponding normal tissues. **C**, *CDH1* mRNA is significantly downregulated in kidney carcinoma (*N* = 100; T = 523) compared to the normal samples. Grey, normal; red, carcinoma. **, *p* < 0.01

The loss of E-cad expression is considered a hallmark of cancer invasion and metastasis potentially via a role in EMT [12]. To determine whether the reported loss of E-cad expression in some cancers [14, 15, 41] is consistent with *CDH1* transcription in clinical tissue samples, we assessed mRNA levels in carcinoma and normal tissues in various parts of the body from the TCGA and GTEx databases using the GEPIA2 web server (http://gepia2.cancer-pku.cn/#index). Surprisingly, most carcinoma tissues did not show significant reductions in *CDH1* mRNA levels compared to normal tissues (Fig. 1). Among the 9 types of commonly occurring carcinomas analyzed, *CDH1* mRNA levels in 6 of them (67%) were upregulated (Fig. 1A), 2 of them (22%) were unchanged (Fig. 1B), and only one of them (11%) was downregulated (Fig. 1C) compared to corresponding normal tissues. Thus, most carcinoma samples examined did not exhibit a reduction in *CDH1* mRNA levels.

**Fig. 2:**
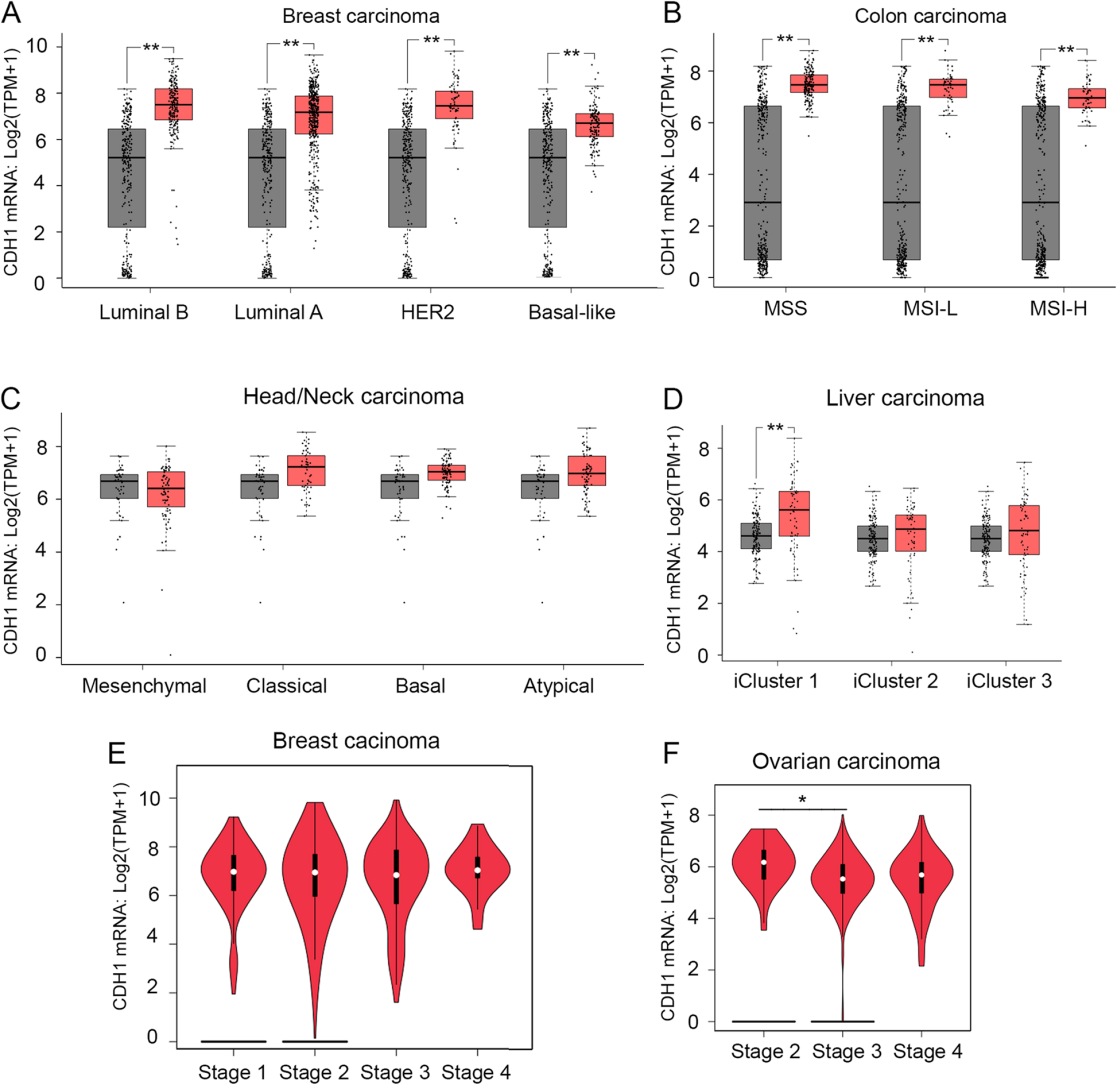
*CDH1* mRNA is either upregulated or remains unchanged in tumor subtypes and major stages of most carcinoma tissues. The levels of *CDH1* mRNA were significantly upregulated in all breast cancer subtypes luminal B (T = 194), luminal A (T = 415), HER2 (T = 66), and basal-like (T = 135) compared to the normal tissues (*N* = 291) (**A**) and in all colon cancer subtypes: MSS (T = 175), MSI-L (T = 48) and MSI-H (T = 48) compared to the normal tissues (*N* = 349) (**B**). **C**, *CDH1* mRNA levels remain unchanged in the four subtypes of head/neck carcinoma subtypes: mesenchymal (T = 75), classical (T = 49), basal (T = 87), and atypical (T = 67) compared to the normal tissues (*N* = 44). **D**, *CDH1* mRNA is significantly upregulated in iCluster 1 (T = 53) but remains unchanged in iCluster 2 (T = 55) and iCluster 3 (T = 63) of liver carcinoma compared to the normal tissues (*N* = 160). Grey, normal; red, carcinoma in (**A**)-(**D**). The levels of *CDH1* mRNA are not significantly different across the major stages of breast carcinoma (*p*-value = 0.265) (**E**) but are significantly downregulated when ovarian carcinoma progresses from stage 2 to stage 3 (*p*-value = 0.015) (**F**). *, *p* < 0.05, **, *p* < 0.01

We then assessed *CDH1* mRNA levels in different subtypes or stages of carcinomas in which *CDH1* mRNA was upregulated or unchanged. The levels of *CDH1* mRNA were significantly upregulated in all subtypes of breast carcinoma (Fig. 2A), colon carcinoma (Fig. 2B), pancreatic carcinoma (Supplementary Fig. 1A—Additional file 1), and lung carcinoma (Supplementary Fig. 1B—Additional file 1) and remained mostly unchanged in the subtypes of head/neck carcinoma and liver carcinoma (Fig. 2C-D). Further analysis demonstrated that the *CDH1* mRNA levels were not significantly changed across the major cancer stages in breast carcinoma (Fig. 2E), colon carcinoma, pancreatic carcinoma, lung carcinoma, and endometrial carcinoma (Supplementary Fig. 1C-F—Additional file 1). Among the cancers examined, the levels of *CDH1* mRNAs only exhibited significant downregulation between stage 2 and stage 3 of ovarian cancer (Fig. 2F). These results suggest that the *CDH1* mRNA upregulation occurs in the early stages of tumor development and the levels remain high as tumors progress to later stages across most carcinoma subtypes except for ovarian cancer, where downregulation of *CDH1* mRNA is observed when the tumors progress from stage 2 to stage 3 (Fig. 2F). Since *CDH1* mRNA is markedly upregulated in some types of tumors, for example, colon carcinoma (26x) and endometrial carcinoma (148x), compared to corresponding normal tissues (Fig. 1A), with further research, *CDH1* mRNA levels may be established as a diagnostic biomarker for early detection of these carcinomas.

### *CDH1* mRNA is highly expressed in most carcinoma cell lines while non-epithelial cancer cell lines exhibit lower expression of *CDH1* mRNA

**Fig. 3:**
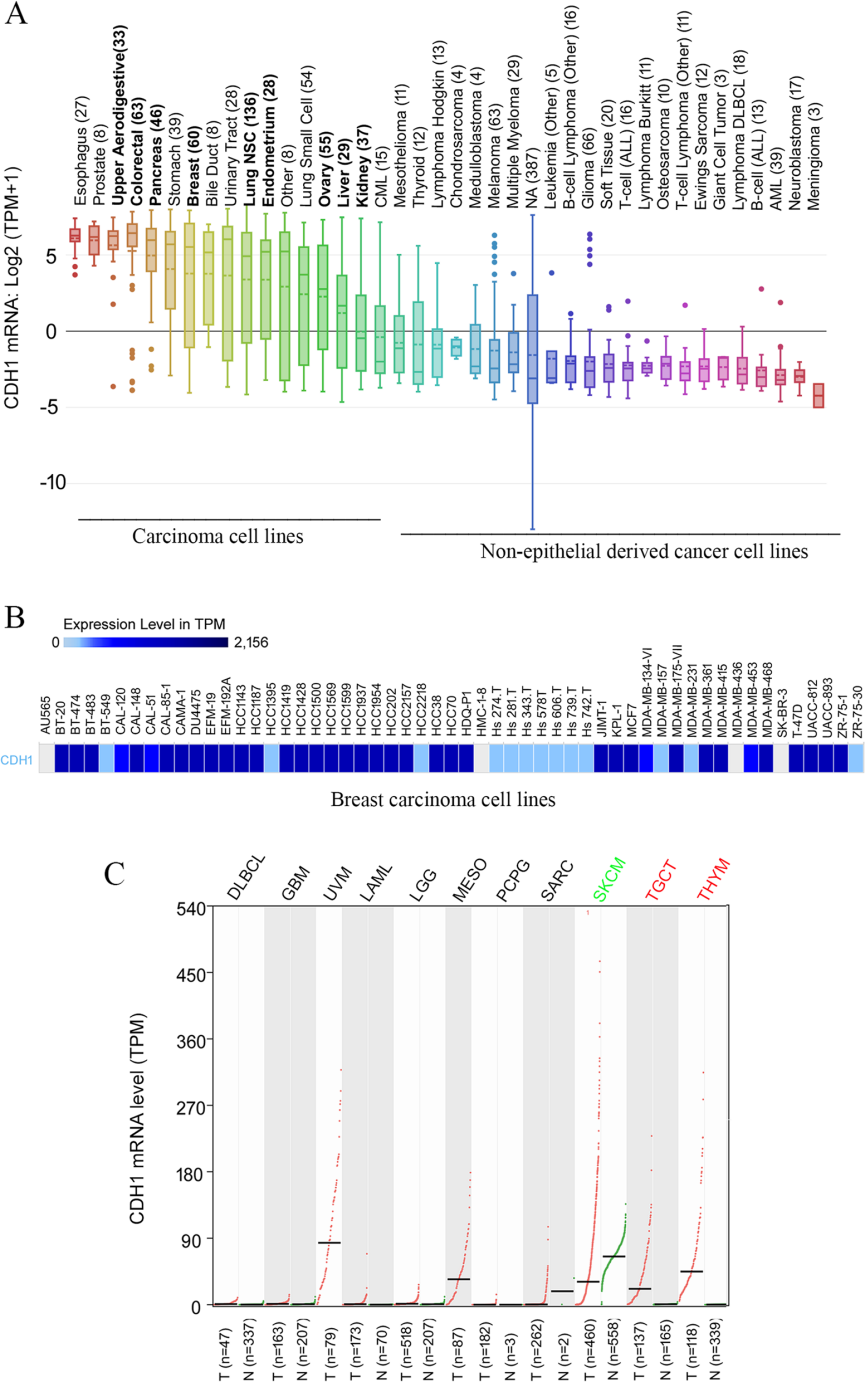
*CDH1* mRNA is highly expressed in most carcinoma cell lines and is expressed at low levels in non-epithelial cancer cell lines. **A**, *CDH1* mRNA is highly expressed in carcinoma cell lines but is expressed at low levels in non-epithelial cancer cell lines. **B**, *CDH1* expression in breast cancer cell lines, shown as a representative of CDH mRNA expression in most carcinoma cell lines. **C**, *CDH1* mRNA levels remain unchanged in most non-epithelial tumor tissues compared to the corresponding normal tissues (cancer name in black) except for skin cutaneous melanoma (SKCM) which demonstrated a significant downregulation (green; *q* < 0.01), and testicular germ cell tumor (TGCT) and thymoma (THYM) which demonstrated an upregulation of *CDH1* mRNA compared to the corresponding normal tissues (red; *q* < 0.01). DLBCL, diffuse large B-cell lymphoma; GBM, glioblastoma multiforme; UVM, uveal melanoma; LAML, acute myeloid leukemia; LGG, brain lower grade glioma; MESO, mesothelioma; PCPG, pheochromocytoma and paraganglioma; SARC, sarcoma

To further assess the transcription of the *CDH1* gene, we analyzed *CDH1* mRNA levels in different cancer cell lines stored on the DepMap portal (https://depmap.org/portal/). Consistent with the data on tumor tissues (Fig. 1), *CDH1* mRNA expression in most carcinoma cell lines derived from commonly occurring carcinomas was in the range of moderate to high (Fig. 3A, Table 1). For example, among 55 breast cancer cell lines, 34 (62%) cell lines exhibited high levels of *CDH1* mRNA, 4 (7%) exhibited medium levels, and 17 (31%) cell lines showed low or no detection of *CDH1* mRNA (Fig. 3B, Table 1).

**Table 1:** *CDH1* mRNA expression in cancer cell lines derived from commonly occurring carcinomas

		***CDH1* mRNA expression**^**a**^		
**Cancer type**	**Number of cancer cell lines**	**High**	**Medium**	**Low/No detection**
Colorectal carcinoma	45	75%	7%	18%
Pancreatic carcinoma	40	63%	22%	15%
Endometrial carcinoma	25	56%	16%	28%
Breast carcinoma	55	62%	7%	31%
Lung carcinoma	69	52%	18%	30%
Ovarian carcinoma	25	32%	16%	52%
Kidney carcinoma	14	14%	14%	72%
Liver carcinoma	22	5%	41%	54%

^a^*CDH1* mRNA expression is expressed as the percentage of the cell lines that express high (101–2,120 TPM), medium (11–100 TPM), or low/no detection (0–10 TPM) levels of *CDH1* mRNA out of the total cell lines for each cell lineage. Data were obtained from the EMBL-EBI Expression Atlas (https://www.ebi.ac.uk/gxa/experiments/E-MTAB-2770/Results) [34]

While E-cad plays an essential role in cell–cell adhesion in epithelial tissues [3, 10, 11, 42], *CDH1* is also expressed in non-epithelial cells [43]. Consistent with its non-essential role in cell–cell adhesion in non-epithelial tissues/cells, *CDH1* levels were lower than those in carcinoma cells (Fig. 3A). In addition, *CDH1* mRNA levels did not significantly change in most of the tested non-epithelial cancers (including lymphoid neoplasm diffuse large B-cell lymphoma, glioblastoma multiforme, acute myeloid leukemia, brain lower-grade glioma, mesothelioma, pheochromocytoma and paraganglioma, sarcoma, and uveal melanoma) compared to corresponding normal tissues (Fig. 3C). The exceptions were testicular germ cell tumors and thymoma, in which *CDH1* mRNA was upregulated, and skin cutaneous melanoma, in which *CDH1* mRNA was downregulated compared to corresponding normal tissue (Fig. 3C).

### E-cad protein is not downregulated in most carcinoma tissues and carcinoma cell lines

**Fig. 4:**
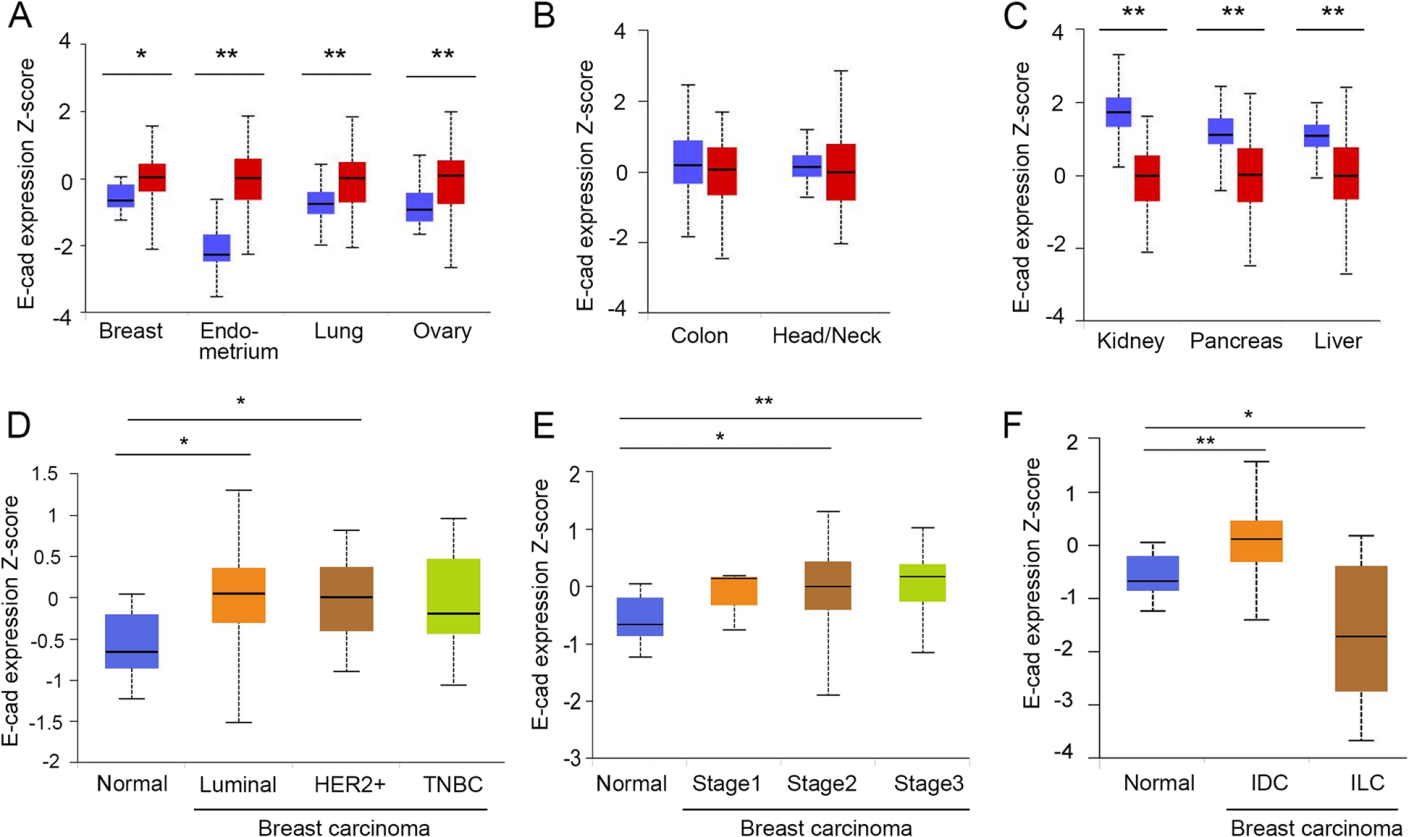
E-cad protein is either significantly upregulated or remains unchanged in most carcinoma tissues. **A**, E-cad protein expression is significantly upregulated in breast carcinoma (*N* = 25; T = 125), endometrial carcinoma (*N* = 31; T = 100), ovarian carcinoma (*N* = 25; T = 100), and lung carcinoma (*N* = 111; T = 111) compared to their corresponding normal tissues. **B**, E-cad protein expression remains unchanged in colon carcinoma (*N* = 100; T = 97) and head/neck carcinoma compared to the corresponding normal tissues (*N* = 71; T = 108). **C**, E-cad protein expression is downregulated in kidney carcinoma (*N* = 84; T = 110), pancreatic carcinoma (*N* = 74; T = 137), and liver carcinoma (*N* = 165; T = 165) compared to their corresponding normal tissues. Blue, normal; red, carcinoma in (**A**)-(**C**). E-cad protein is significantly upregulated in the different subtypes: luminal (*n* = 64), HER2 + (*n* = 10), and TNBC (*n* = 16) (**D**), and in the major stages: stage 1 (*n* = 4), stage 2 (*n* = 74), and stage 3 (*n* = 32) (**E**) of breast carcinomas compared to the normal tissues (*n* = 18). **F**, E-cad expression is significantly upregulated in IDC (*n* = 93) but downregulated in ILC (*n* = 10) compared to the normal tissues (*n* = 18). *, *p* < 0.05, **, *p* < 0.01

We next determined how E-cad protein changed in carcinoma tissues since higher mRNA levels do not always result in higher protein levels [44–49]. Consistent with *CDH1* mRNA expression in most carcinoma tissues (Fig. 1) and carcinoma cell lines (Fig. 3A-B), E-cad protein was either significantly upregulated or remained unchanged in most carcinoma tissues compared to corresponding normal tissue (Fig. 4A-B). Among the tested carcinoma tissues, E-cad levels were significantly upregulated in breast, endometrial, ovarian, and lung carcinomas (Fig. 4A), and remained unchanged in colon and head/neck carcinomas (Fig. 4B); the levels of E-cad were significantly downregulated only in kidney, pancreatic, and liver carcinomas (Fig. 4C).

In carcinomas in which E-cad was upregulated/unchanged, this expression pattern was reflected in most cancer subtypes and stages (Fig. 4E and Supplementary Fig. 2—Additional file 1). For example, E-cad expression was significantly upregulated in luminal (estrogen receptor (ER) + , progesterone receptor (PR) ± , human epidermal growth factor 2 (HER2)-, and low levels of Ki-67 protein), and HER2 positive breast cancer (ER-, PR-, and HER2 +), and remained unchanged in triple-negative breast cancer (ER-, PR-, HER2-) (TNBC) compared to normal tissues (Fig. 4D). In addition, E-cad levels were mostly upregulated or remained unchanged during tumor progression in most carcinomas (Table 2). For example, the levels of E-cad were significantly upregulated in stage 2, and stage 3 and remained unchanged in stage 1 of breast carcinoma compared to normal tissue (Fig. 4E). Similarly, E-cad levels were predominately upregulated or remained unchanged in the major cancer stages of endometrial, lung, ovarian, head/neck, and colon carcinomas (Supplementary Fig. 2—Additional file 1). Interestingly, E-cad is significantly downregulated in infiltrating lobular carcinoma (ILC) of breast cancer, consistent with the observations from other groups [14, 50–52], but upregulated in infiltrating ductal carcinoma (IDC) (Fig. 4F), suggesting that E-cad levels may serve as a marker to differentiate ILC from IDC breast cancer.

**Table 2:** E-cad protein expression in carcinoma tissues compared to the corresponding normal tissues

	**E-cad protein expression**^**a**^			
**Carcinoma type**	**Stage 1**	**Stage 2**	**Stage 3**	**Stage 4**
Breast carcinoma	Unchanged (4)	Up (74)	Up (32)	--------------
Endometrial carcinoma	Up (74)	Up (8)	Up (15)	Unchanged (3)
Ovarian carcinoma	Up (2)	--------------	Up (75)	Up (16)
Lung carcinoma	Up (59)	UP (30)	Up (21)	--------------
Colorectal carcinoma	Unchanged (10)	Unchanged (39)	Unchanged (40)	Unchanged (8)
Head/Neck carcinoma	Down (7)	Unchanged (25)	Unchanged (30)	Unchanged (46)
Kidney carcinoma	Down (52)	Down (13)	Down (33)	Down (12)
Pancreatic carcinoma	Down (4)	Down (65)	Down (35)	Down (7)

^a^Numbers in the parentheses are the number of samples in each disease stage. The number of normal samples used are: breast, 18; endometrium, 31; ovary, 25; lung, 59; colon, 10; head/neck, 7; kidney, 52; pancreas, 4. Data were obtained from the CPTAC via the UALCAN portal (http://ualcan.path.uab.edu/analysis-prot.html) and was analyzed using t-test on log2-spectral count ratios [37, 40]

In agreement with the data from tissues, analysis of the CCLE proteomics database in the DepMap portal revealed that most of the tested carcinoma cell lines expressed elevated levels of E-cad protein. Specifically, 67% of breast carcinoma cell lines, 77% of colorectal carcinoma cell lines, 60% of pancreatic carcinoma cell lines, 77% of endometrial carcinoma cell lines, and 100% of head/neck carcinoma cell lines expressed elevated levels of E-cad. Most of the cell lines derived from kidney, liver, lung, and ovarian carcinomas exhibited low levels of E-cad (Table 3).

**Table 3:** E-cad protein expression in cancer cell lines derived from commonly occurring carcinomas

		**E-cad protein expression**^**a**^	
**Carcinoma type**	**Number of cell lines**	**High**	**Low**
Breast carcinoma	30	67%	33%
Colorectal carcinoma	30	77%	23%
Pancreatic carcinoma	20	60%	40%
Endometrial carcinoma	13	77%	23%
Head/Neck carcinoma	10	100%	0%
Kidney carcinoma	12	8%	92%
Liver carcinoma	14	36%	64%
Lung carcinoma	79	37%	63%
Ovarian carcinoma	17	41%	59%

^**a**^E-cad expression is expressed as the percentage of the cell lines that express high or low levels of E-cad out of the total cell lines for each cell lineage. Data were obtained from the DepMap portal (https://depmap.org/portal/interactive/); high is defined as log2-transformed values higher than 0 and low expression was defined as log2-transformed values lower than 0 [38]

**Fig. 5:**
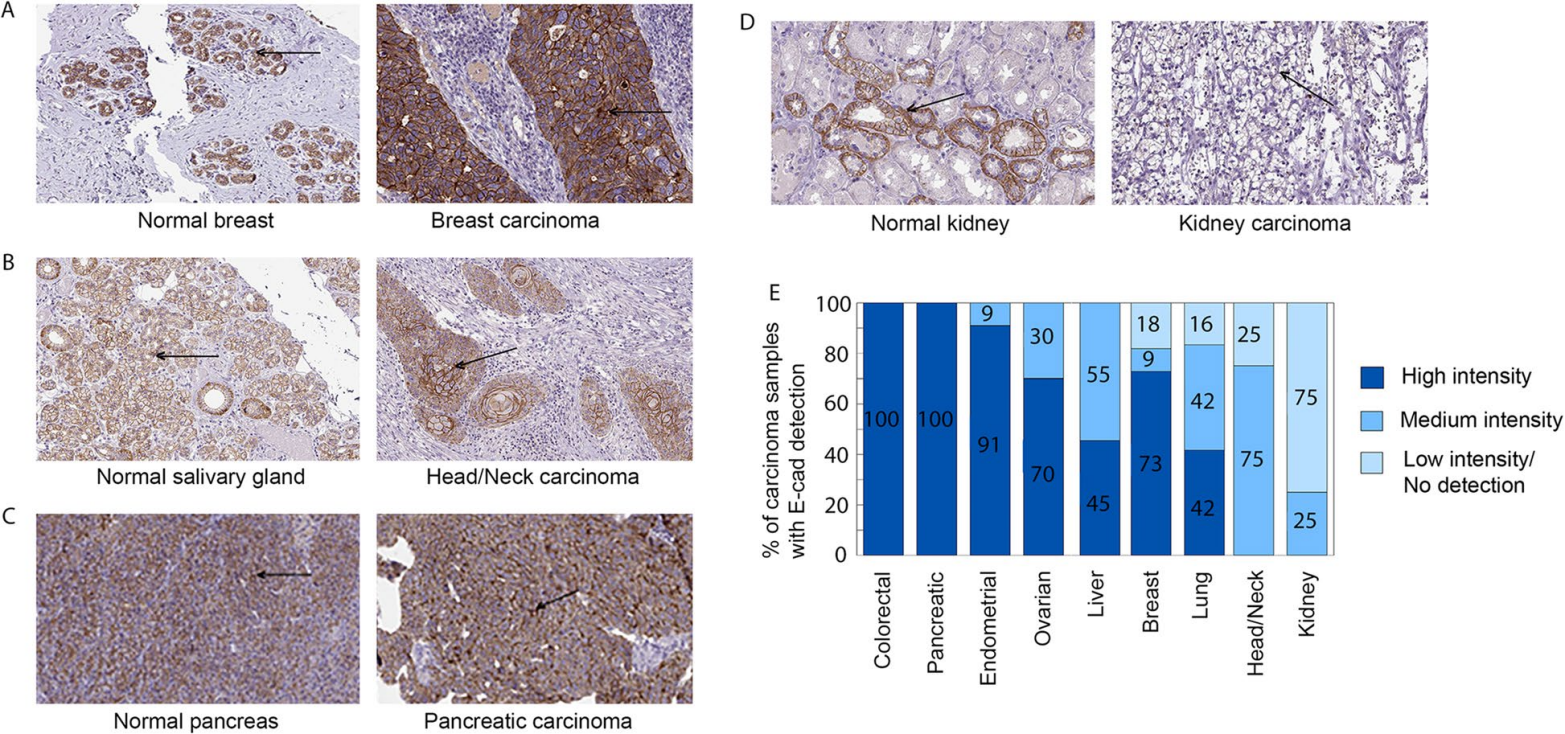
Immunohistochemistry staining reveals that E-cad protein is not downregulated in most carcinoma tissues. Immunohistochemistry staining of E-cad in breast carcinoma (**A**), head/neck carcinoma (**B**), pancreatic carcinoma (**C**), and kidney carcinoma (**D**), and their corresponding normal tissues. **E**, Most carcinoma tissues exhibit medium to high intensity of E-cad staining except for kidney carcinoma, which exhibits little or no E-cad staining in most of the tumor tissues

We also assessed the scanned images of E-cad immunohistochemistry staining of different carcinoma tissues stored on the HPA website (https://www.proteinatlas.org/humanproteome/pathology) [39]. All the representative samples for colorectal, pancreatic, endometrial, ovarian, and liver carcinomas, 82% of breast cancer, 84% of lung cancer, and 75% of head/neck carcinoma samples exhibited medium to high levels of E-Cad staining, which were comparable to those of corresponding normal tissues (Fig. 5A-C, and E). Kidney carcinoma was the only tumor that showed lower E-cad staining compared to normal tissues (Fig. 5D-E), consistent with its *CDH1* mRNA expression profile (Fig. 1C). These immunohistochemistry staining results are also consistent with the E-cad protein expression profiles revealed by the mass spectrometry analysis, which demonstrated that E-cad expression was upregulated or remained unchanged in most carcinoma tissues (Fig. 4A-B) and was expressed at higher levels in most of the carcinoma cell lines analyzed (Table 3).

### E-cad protein is not downregulated when most primary carcinomas progress to metastatic tumors

**Fig. 6:**
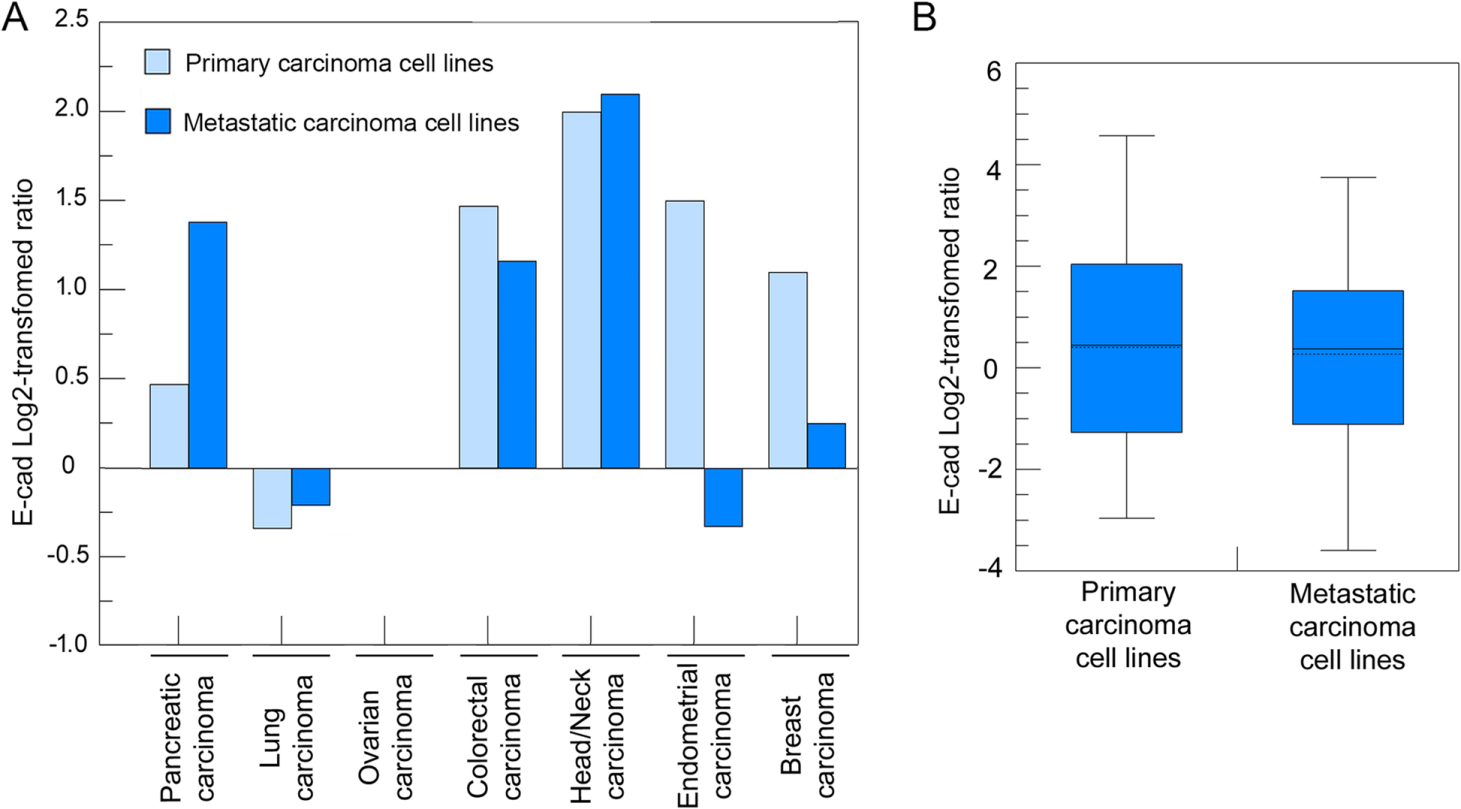
E-cad protein levels are not significantly changed in the carcinoma cell lines derived from metastatic tumors compared to the carcinoma cell lines derived from primary tumors. **A**, E-cad protein levels are not significantly different in the carcinoma cell lines derived from metastatic tumors compared to the carcinoma cell lines derived from primary tumors of pancreatic (11 metastatic (M); 7 primary (P)), lung (49 M; 30 P), ovarian (8 M; 9 P), colorectal (9 M; 17 P), head/neck (4 M; 6 P), endometrial (2 M; 11 P), and breast (16 M; 14 P) carcinomas. **B**, The E-cad level in the combined metastatic carcinoma cell lines was not statistically different from the level in the combined primary tumor cell lines (101 M; 117 P)

Since the E-cad protein is postulated to play a critical role during the transition from primary tumors to metastatic tumors in carcinomas [12, 13, 28, 53, 54], we assessed how the E-cad levels differed in the cell lines derived from metastatic tumors compared to those derived from primary tumors using the proteomics data on the CCLE available in the DepMap portal. The levels of E-cad were not significantly different between the metastatic cell lines and primary cells lines in any of the specific lineages (Fig. 6A), nor were they significantly different when all the metastatic cell lines were compared to all the primary tumor cell lines encompassing all 9 lineages (Fig. 6B). Thus, on average, the levels of E-cad protein in metastatic carcinoma cell lines remained unchanged compared to primary tumor cell lines, suggesting that E-cad protein is not downregulated in commonly occurring carcinomas when primary tumors progress to metastatic tumors. It is also possible that the cells are able to revert back to an epithelial phenotype after settling down in the new sites due to the MET process.

### *CDH1* mRNA and E-cad protein are indeed downregulated in certain cancers

**Fig. 7:**
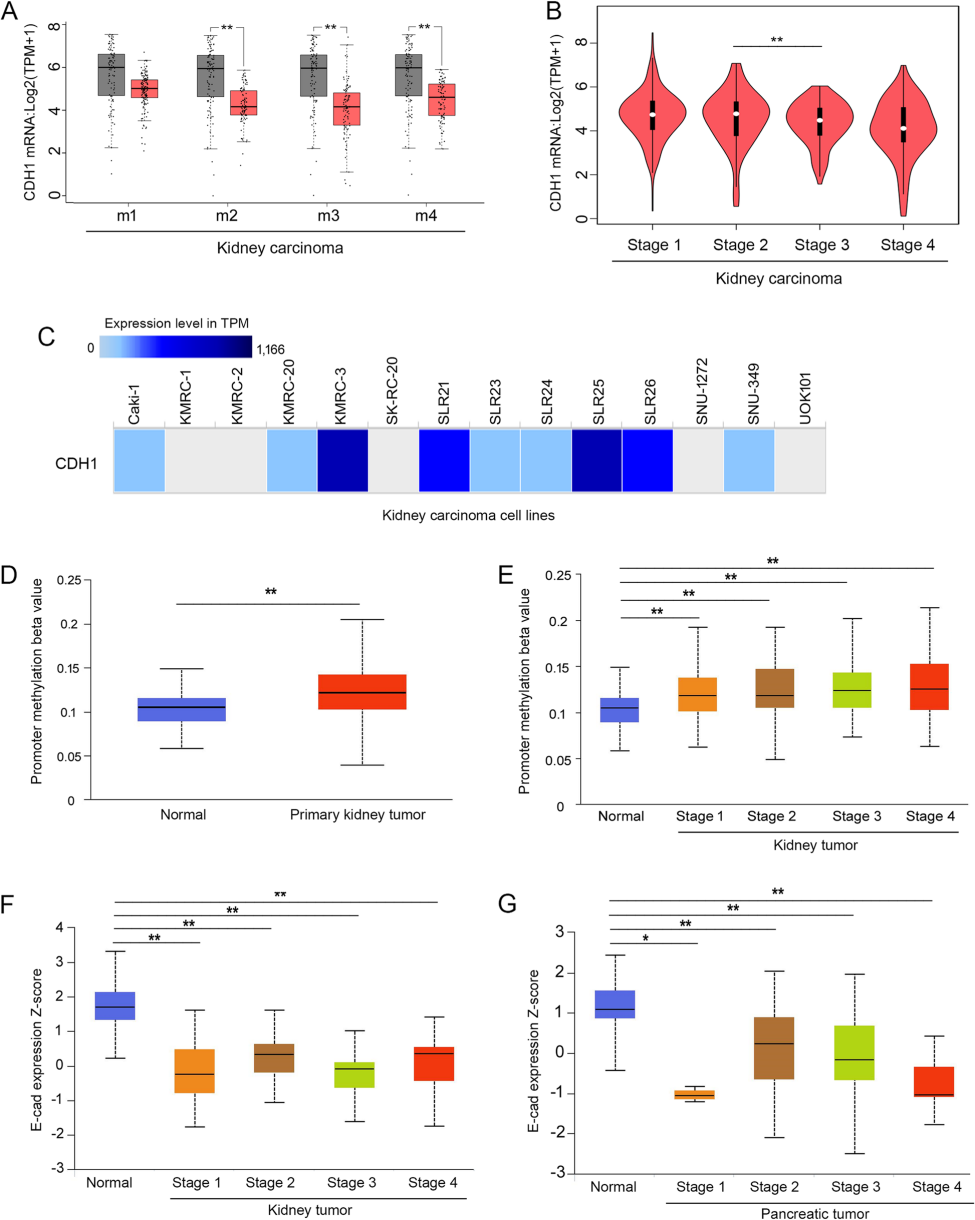
*CDH1* mRNA is downregulated in kidney tumors and the downregulation potentially results from promoter methylation. **A**, The *CDH1* mRNA levels are significantly decreased in 3 of the 4 subtypes of kidney cancer: m2 (*n* = 85), m3 (*n* = 93), and m4 (*n* = 85) and remain unchanged in m1 (*n* = 145) compared to the normal tissues (*n* = 100) (grey, normal; red, carcinoma). **B**, *CDH1* mRNA is significantly downregulated across the major stages of kidney carcinoma with a decrease in *CDH1* mRNA between stage 2 and stage 3 (*p*-value = 0.00025). **C**, Most kidney carcinoma cell lines exhibit low or no *CDH1* mRNA expression. **D**, *CDH1* promoter methylation in kidney carcinoma (*n* = 324) is significantly higher than in the normal tissues (*n* = 160). **E**, *CDH1* promoter methylation is significantly increased in stage 1 (*n* = 160), stage 2 (*n* = 31), stage 3 (*n* = 73), and stage 4 (*n* = 58) compared to the normal tissues (*n* = 160). **F**, E-cad protein expression is significantly decreased in stage 1 (*n* = 52), stage 2 (*n* = 13), stage 3 (*n* = 33), and stage 4 (*n* = 12) of kidney carcinoma compared to the normal tissues (*n* = 84). **G**, E-cad protein expression is significantly decreased in stage 1 (*n* = 4), stage 2 (*n* = 85), stage 3 (*n* = 35), and stage 4 (*n* = 7) of pancreatic cancer compared to the normal tissues (*n* = 74). *, *p* < 0.05, **, *p* < 0.01

Suppression of *CDH1* gene expression in cancer cells has been widely reported [55–58], particularly in cancer cell line-based studies [41, 59, 60]. Indeed, we found that the levels of *CDH1* mRNA and E-cad protein were downregulated in certain carcinomas, particularly kidney carcinoma (Figs. 1C and 4C). When kidney carcinoma was divided into four subtypes based on the differential expression of 500 genes and 500 microRNAs [61], *CDH1* mRNA levels were significantly downregulated in cluster 2 (m2), cluster 3 (m3), and cluster 4 (m4), but remained unchanged in cluster 1 (m1) compared to normal tissues (Fig. 7A). In addition, the *CDH1* mRNA levels were significantly downregulated during tumor progression in kidney tumors, with lower levels in the advanced stage 3, in which tumors have intruded into veins and lymph nodes, and the lowered levels of *CDH1* mRNA persisted in stage 4, in which tumors have grown in tissues outside the kidney and in distant organs (Fig. 7B) [62]. The decreased levels of *CDH1* mRNA in advanced stages coincide with the invasion of surrounding vessels and tissues, suggesting that the lower levels of *CDH1* mRNA in kidney carcinoma are closely related to tumor invasion. These results are consistent with the *CDH1* mRNA levels in the kidney carcinoma cell lines, which were at the lower end of the levels in the carcinoma cell lines examined (Fig. 3A). In addition, 10 out of the 14 examined kidney carcinoma cell lines (71.4%) exhibited low or no detection of *CDH1* mRNA, and only 4 out of the 14 (28.6%) kidney carcinoma cell lines exhibited moderate to high levels of *CDH1* mRNA (Fig. 7C and Table 1).

Promoter methylation is a major mechanism in suppressing tumor suppressor gene expression [55–58]. We examined the *CDH1* promoter methylation using the UALCAN web server (http://ualcan.path.uab.edu/analysis.html). *CDH1* promoter methylation was significantly higher in kidney primary tumors than in normal tissues (Fig. 7D). In addition, *CDH1* promoter methylation is significantly elevated in all stages of kidney carcinoma compared to normal samples (Fig. 7E). These results demonstrate that promoter methylation plays a critical role in suppressing the transcription of the *CDH1* gene in kidney carcinoma.

Consistent with the lower levels of *CDH1* mRNAs in kidney carcinoma (Fig. 7A-C), analysis using the UALCAN web server revealed that the levels of E-cad protein in kidney cancer were significantly downregulated in tumors compared to normal tissue (Fig. 4C) and E-cad was not detectable in the immunohistochemistry staining of kidney carcinoma (Fig. 5D), demonstrating that the lower levels of *CDH1* mRNA result in lower levels of E-cad protein [37]. This relationship suggests that the expression of E-cad protein in kidney carcinoma is regulated at the transcription level, most likely due to CpG methylation (Fig. 7D-E), allelic deletion of 16q22.1 containing the E-cad locus, or nonsense mutations [14]. E-cad protein levels were also significantly decreased in all stages of kidney carcinoma compared to normal tissue (Fig. 7F), suggesting that the loss of E-cad expression occurs initially in the early stages of tumor development and is maintained through later stages.

Decreased levels of E-cad protein were also detected in pancreatic carcinoma (Fig. 4C). The downregulation of E-cad protein was observed in all stages of pancreatic carcinoma compared to the normal tissue (Fig. 7G). These results are intriguing because transcriptomics data revealed upregulation of *CDH1* mRNA in pancreatic carcinoma tissues compared to normal tissue (Fig. 1A) and higher *CDH1* mRNA levels in pancreatic cancer cell lines compared to other carcinoma cell lines (Fig. 3A). The E-cad immunohistochemistry staining also revealed that the E-cad protein was expressed at elevated levels in all the pancreatic carcinoma samples analyzed (Fig. 5C). The conflicting results warrant more careful examinations of *CDH1* mRNA levels and E-cad protein levels in pancreatic cancer tissues and cell lines in the future.

### *CDH1* mRNA levels and E-cad protein levels in carcinomas are positively correlated, and the *CDH1* mRNA levels are correlated to cancer patient’s survival

**Fig. 8:**
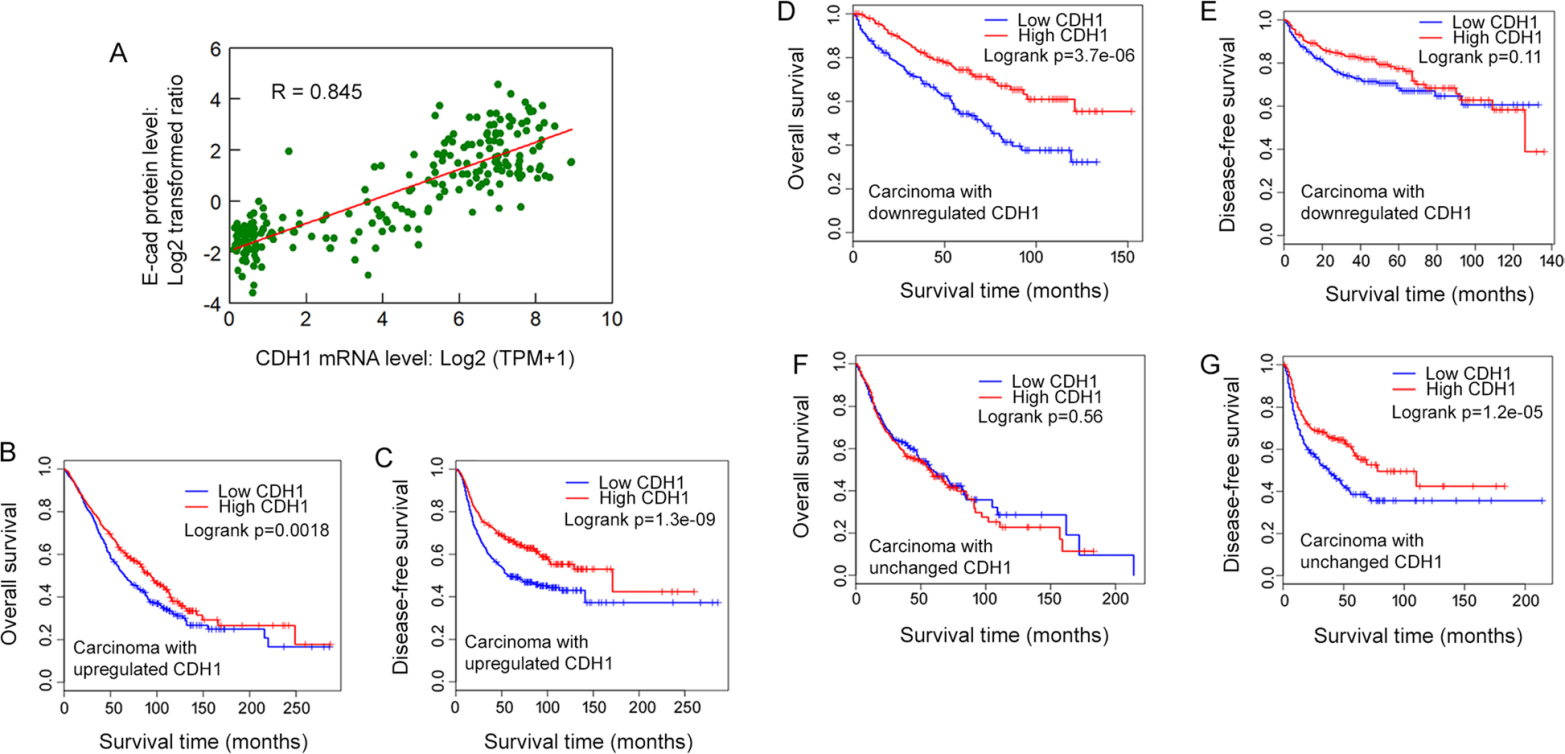
Positive correlation between *CDH1* mRNA levels and E-cad protein levels in carcinoma cell lines. **A**, The *CDH1* mRNA levels and E-cad protein levels in carcinoma cell lines are positively correlated (*R* = 0.845). *CDH1* mRNA and E-cad protein data from tumor cell lines derived from the endometrium (*n* = 14), head/neck (*n* = 10), pancreas (*n* = 19), ovary (*n* = 17), lung (*n* = 77), liver (14), kidney (*n* = 12), colon (*n* = 29), and breast (*n* = 30) were used in the plot. *CDH1* mRNA and E-cad data were obtained from Expression 22Q1 Public and Proteomics datasets in the DepMap portal (https://depmap.org/portal/). **B** and **C**, OS and DFS of breast, colon, lung, pancreatic, ovarian, and endometrial carcinoma patients who carry tumors with upregulated *CDH1* mRNA (High = 1,298; Low = 1,298) based on *CDH1* mRNA expression. **C** and **D**, OS and DFS of kidney carcinoma patients who carry tumors with downregulated *CDH1* (High = 258; Low = 258) based on *CDH1* mRNA expression. **F** and **G**, OS and DFS of liver and head/neck carcinoma patients who carry tumors with the unchanged expression of *CDH1* (High = 441; Low = 441) based on *CDH1* mRNA expression

The relationship between the levels of *CDH1* mRNA and E-cad protein in the carcinoma cell lines from nine different lineages stored on DepMap portal was analyzed using Pearson correlation analysis and Spearman correlation analysis. *CDH1* mRNA levels were strongly positively correlated with E-cad protein levels for eight of the nine lineages of carcinoma cell lines (Table 4). The exception was the kidney carcinoma lineage, in which the cell lines exhibited a weak correlation between *CDH1* mRNA levels and E-cad protein levels (Table 4). When all the cell lines were analyzed together, *CDH1* mRNA levels and E-cad levels exhibited a strong positive correlation (Fig. 8A). These results suggest that, at least at the cell line level, the expression of E-cad is regulated at the transcriptional level in most carcinoma cell types. To determine whether the same observation can be made at the tissue level, a database with proteomics and transcriptomics analyses of carcinoma tissue samples would be beneficial.

**Table 4:** Pearson correlation analysis and Spearman correlation analysis of E-cad levels and *CDH1* mRNA levels in carcinoma cell lines

Carcinoma type	Number of cell lines	Pearson	Spearman	Slope	Intercept
Pancreatic carcinoma	19	0.78	0.80	5.59E-01	-2.00E + 00
Ovarian carcinoma	17	0.81	0.84	5.52E-01	-1.92E + 00
Lung carcinoma	77	0.84	0.81	4.93E-01	-2.13E + 00
Liver carcinoma	14	0.93	0.84	5.59E-01	-1.83E + 00
Kidney carcinoma	12	0.25	0.23	4.02E-01	-1.49E + 00
Head/neck carcinoma	10	0.73	0.75	1.03E + 00	-4.68E + 00
Endometrial Carcinoma	14	0.94	0.71	5.96E-01	-1.74E + 00
Colorectal Carcinoma	29	0.77	0.65	5.33E-01	-1.91E + 00
Breast carcinoma	30	0.80	0.66	4.83E-01	-1.98E + 00

The data were obtained from the DepMap Portal; for the E-cad protein expression, the Proteomics dataset was used [38], and for the *CDH1* mRNA dataset, Expression 22Q1 Public was used [33]. The data were obtained from the DepMap Portal: (https://depmap.org/portal/interactive/?filter=&regressionLine=false&associationTable=false&x=slice%2Fexpression%2F3962%2Fentity_id&y=slice%2Fproteomics%2F246639%2Fentity_id&color=slice%2Flineage%2F2%2Flabel)

We performed cancer patient survival analysis to assess whether the *CDH1* mRNA expression is correlated to cancer patient’s survival. For the patients with carcinomas that had upregulated *CDH1* mRNA, including endometrial, pancreatic, ovarian, lung, colon, and breast carcinomas (Fig. 1A), cancer patients with tumors that expressed higher levels of *CDH1* mRNA (*n* = 1,298) fared significantly better than those with tumors that expressed lower levels of *CDH1* mRNA (*n* = 1,298) with better OS and DFS (*p* = 0.0018 and 1.3E-09, respectively) (Fig. 8B-C). For the patients with carcinomas that had downregulated *CDH1* mRNA, including kidney cancer (Fig. 1C), *CDH1* mRNA expression had a significant effect on OS but not DFS (High, *n* = 258 vs Low, *n* = 258; *p* = 3.7E-06 and 0.11, respectively) (Fig. 8D-E). For the patients diagnosed with carcinomas that had unchanged levels of *CDH1* mRNA compared to normal tissues, including liver and head/neck carcinomas (Fig. 1B), tumor *CDH1* mRNA levels were not significantly correlated to OS but were significantly correlated to DFS (High, *n* = 441 vs Low, *n* = 441; *p*, = 0.56 and 1.2E-05, respectively) (Fig. 8F-G). Overall, these results demonstrate that higher levels of *CDH1* mRNA expression are correlated with better survival of carcinoma patients.

## Discussion

The loss of E-cad has been widely considered a hallmark of metastatic cancers and critical for metastasizing tumor cells to break away from the epithelial tissues to invade the tumor stroma [12–14]. This observation was established primarily with the help of invasive lobular breast cancer (ILC) tissues, in which the loss of E-cad has been shown to play a key role [14, 50–52]. Our analysis of clinical cancer tissues revealed that *CDH1*/E-cad expression was downregulated only in a few types or subtypes of tumors among the large group of tumor types or subtypes examined; ILC happens to be a subtype of breast tumors in which E-cad expression was downregulated (Fig. 4F). Another major exception is kidney carcinoma, which exhibited the well-described loss of E-cad expression (Figs. 1C and 7). In agreement with recent debates on the role of E-cad in tumor progression and metastasis [63, 64], our analysis demonstrates that *CDH1* mRNA and E-cad protein are not downregulated in the majority of carcinomas (Figs. 1, 4, and 5) or during tumor progression in most carcinomas (Figs. 2 and 4E, and Supplementary Figs. 1 and 2—Additional file 1). For a more detailed analysis of the role of E-cad in EMT in tumor progression, tumor samples exhibiting hybrid EMT or partial EMT [65–68] may be required. Because it is difficult to obtain data on the hybrid EMT or partial EMT samples from cancer patients, at least in large quantities, our studies cannot provide insight into the role of E-cad in complex phenomena like hybrid EMT or partial EMT in metastasis, which shows a limitation of this type of study.

It is interesting to note that *CDH1* mRNA and/or E-Cad were upregulated in most cancers in the early stages of tumor development and the levels remained elevated as tumors progressed to later stages across most carcinoma types (Figs. 2 and 4E, and Supplementary Figs. 1 and 2—Additional file 1). These results suggest that most carcinomas may require higher levels of E-cad expression for tumor formation and tumor progression in earlier stages of tumor development, and this requirement needs to be maintained even after metastasis has occurred. One possibility is that the upregulation of *CDH1*/E-cad expression in carcinoma cells is an adaptive response to the abnormal signaling inside tumor cells, which is known to result in increasingly altered cell–cell adhesion and actin cytoskeleton rearrangement during tumor formation, progression, and invasion [69–71]. For example, it has been shown that tumor cells can upregulate proteins that are directly related to the rearrangement of the actin cytoskeleton [72, 73] and that there is rearrangement (but not loss) of E-cad-based adherens junctions during neoplastic transformation [69]. Tumor cells may respond to these types of changes in cell–cell adhesion and actin cytoskeleton rearrangement by expressing more E-cad to restore the altered cell–cell adhesion and epithelial tissue integrity during tumor formation, progression, and invasion. It has also been shown that E-cad plays an important role in preventing anoikis, the induction of apoptosis after the loss of attachment to the ECM and neighboring cells [71, 74]. To prevent anoikis induced by truncation of the cytoplasmic domain of E-cad which results in disruption of the binding of the domain to β-catenin, a linker protein that connects the actin cytoskeleton to the cytoplasmic domain of E-cad [74, 75], tumor cells may be required to upregulate E-cad [76, 77].

Since *CDH1*/E-cad upregulation is widespread in carcinomas (Figs. 1, 4, and 5) and the levels remain elevated as tumors progressed to later stages across most carcinoma types (Figs. 2 and 4E, and Supplementary Figs. 1 and 2—Additional file 1), the effect of higher levels of *CDH1* mRNA on carcinoma patient’s survival (Fig. 8) suggest that the role of E-cad on carcinoma development and progression is more complex than previously thought and warrants further investigation. Although the Log-rank tests allowed us to establish a positive correlation between *CDH1* mRNA levels and cancer patient’s survival (Fig. 8), the survival tests did not allow us to conclude whether *CDH1* expression is functionally linked to cancer patient’s survival, showing another limitation of this study. To gain further insight into the potential value of *CDH1* mRNA levels in cancer prognosis and the role of *CDH1*/E-cad in carcinoma development and progression, future studies should consider additional clinical data, such as median survival time, age, and tumor stages. In addition, since *CDH1* mRNA is markedly upregulated in some types of tumors, such as colon and endometrial carcinoma (Fig. 1A), from the early stages of tumor development (Fig. 4E, and Supplementary Fig. 2A-C—Additional file 1), it is worth further investigation to determine whether *CDH1* mRNA levels can serve as a reliable biomarker for early diagnosis of these carcinomas.

It is well established that metastatic carcinoma cells invade the stroma and migrate in single cells or collectively in groups [78]. In single-cell invasion/migration, single cells acquire the ability to break away from the primary tumor tissues through the loss of E-cad [79, 80]. In contrast, in collective cell invasion/migration, most of the tumor cells localized in the interior of a cell cluster maintain elevated levels of E-cad expression and only the tumor cells on the edge of the cluster express low levels of E-cad, which allow the cluster of cells to break away from the primary carcinoma tissues [23, 27, 81, 82]. Most previous studies designed to investigate tumor EMT and metastasis normally used *in-vitro* 2-dimensional cell culture (2D) or 3D scaffold cell culture with a focus on single-cell invasion/migration [53, 59, 83, 84]. Results from these types of studies may not reflect the situation in the collective cell invasion/migration [63, 64, 85, 86]. Our findings that *CDH1*/E-cad expression is not significantly downregulated when primary tumors progress into metastatic tumors (Fig. 6), which are consistent with the observations from other groups [13, 20, 28, 29], suggest that single-cell invasion/migration may not be the preferred mode of invasion/migration, and collective invasion/migration might be the predominant form of invasion/migration for most carcinomas, a notion that is supported by several studies monitoring metastatic tumors in circulation [81, 87, 88]. Furthermore, after metastatic carcinoma cells settle down in a new place, the metastatic carcinoma cells re-acquire epithelial cell phenotypes via MET [89, 90]. It is also possible that MET can contribute to the elevated or unchanged levels of E-cad in metastatic cancer cells.

## Conclusion

*CDH1* mRNA and E-cadherin protein are not downregulated in most carcinoma tissues and carcinoma cell lines tested in this study. Thus, the role of E-cad in tumor progression and metastasis may have previously been oversimplified. Because *CDH1* mRNA is markedly upregulated in the early stages of tumor development of some types of tumors, such as colon and endometrial carcinomas, *CDH1* mRNA levels may serve as a reliable biomarker for the early diagnosis of these carcinomas.

## Supplementary Information

**Additional file 1: Supplementary Figure 1. ***CDH1 *mRNAis significantly upregulated in some carcinoma subtypes and remains unchanged across major tumor stages of most carcinomas. *CDH1 *mRNA levels are significantly upregulated in the two subtypes of pancreatic carcinoma: classical (T=86) and basal (T=65) compared to the normal tissues (*N*=171) (A) and in the three subtypes of lung carcinoma: proximal inflammatory (T=78), proximal proliferative (T=58), and terminal respiratory unit (T=68) compared to the normal tissues (*N*=347) (B). Grey, normal; red, carcinoma in (A) and (B). The levels of *CDH1 *mRNA remain unchanged across the major stages of colon (C), pancreatic (D), lung (E), and endometrial (F) carcinomas. **, *p *< 0.01. **Supplementary** **Figure 2.** E-cad protein is either upregulated or remains unchanged in most major tumor stages. E-cad levels are either significantly upregulated or remain unchanged in distinct stages of endometrial carcinoma (A), lung carcinoma (B), ovarian carcinoma (C), head/neck carcinoma (D), and colon carcinoma (E and F) except for stage 1 of head/neck carcinoma (D) compared to the corresponding normal tissues. Numbers of tissues used in the analysis are: endometrial carcinoma: normal, *n*=31, stage 1,n=74, stage 2, *n*=8, stage 3, *n*=15; lung cancer: normal, *n*=11, stage 1, *n*=59, stage 2, *n*=30, stage 3, *n*=21; ovarian cancer: normal, *n*=25, stage 1, *n*=2, stage 3, *n*=75, stage 4, *n*=16; head/neck carcinoma: normal, *n*=71, stage 1, *n*=7, stage 2, *n*=25, stage 3, *n*=75, stage 4, *n*=4; colon carcinoma: normal, *n*=100, mucinous, *n*=19, non-mucinous, *n*=77, stage 1, *n*=10, stage 2, *n*=39, stage 3, *n*=40, stage 4, *n*=8. *, *p *< 0.05, **, *p *< 0.01.

**Additional file 2: Supplementary Table 1.** List of carcinoma cell lines used for CDH1 mRNA expression analysis in Table 1. **Supplementary** **Table 2.** List of carcinoma cell lines used for E-cad expression analysis in Table 3. **Supplementary** **Table 3.** List of carcinoma cell lines used for Pearson analysis and Spearman analysis in Table 4. **Supplementary** **Table 4.** List of cancer cell lines used for CDH1 mRNA expression analysis across different types of cancers in Fig. 3A. **Supplementary Table 5.** List of carcinoma cell lines used for E-cad proteomics analysis comparing metastic vs primary tumor derived cells lines in Fig. 6. **Supplementary** **Table 6.** List of carcinoma cell lines used for the E-cad and CDH1 mRNA correlation analysis in Fig. 8A.

## Data Availability

The datasets analyzed during the current study are available in the Gene Expression Profiling Interactive Analysis 2 (GEPIA2) (http://gepia2.cancer-pku.cn/#index), the University of Alabama at Birmingham Cancer Data Analysis (UALCAN) portal. (http://ualcan.path.uab.edu/analysis-prot.html and http://ualcan.path.uab.edu/analysis.html), the Human Protein Atlas (HPA) (https://www.proteinatlas.org/), and the Cancer Dependency Map (DepMap) portal (https://depmap.org/portal/interactive/).

## Abbreviations

CCLE: Cancer Cell Line Encyclopedia
DepMap: Cancer Dependency Map
E-cad: E-cadherin
DFS: Disease-free survival
OS: Overall survival
ECM: Extracellular matrix
EMT: Epithelial-mesenchymal transition
GEPIA2: Gene Expression Profiling Interactive Analysis 2
GTEx: Genome-Typing Expression
TPM: Transcripts per million
TCGA: The Cancer Genome Atlas
UALCAN: The University of Alabama at Birmingham Cancer Data Analysis Portal
