# Supplementary material for: A comprehensive analysis of different types of databases reveals that CDH1 mRNA and E-cadherin protein are not downregulated in most carcinoma tissues and carcinoma cell lines
Source: BMC Cancer. 2023 May 15;23:441. doi: 10.1186/s12885-023-10916-0 (PMC10184369; doi:10.1186/s12885-023-10916-0)
Supplement: Supplementary file 1 — Additional file 1: Supplementary Figure 1. CDH1 mRNAis significantly upregulated in some carcinoma subtypes and remains unchanged across major tumor stages of most carcinomas. CDH1 mRNA levels are significantly upregulated in the two subtypes of pancreatic carcinoma: classical (T=86) and basal (T=65) compared to the normal tissues (N=171) (A) and in the three subtypes of lung carcinoma: proximal inflammatory (T=78), proximal proliferative (T=58), and terminal respiratory unit (T=68) compared to the normal tissues (N=347) (B). Grey, normal; red, carcinoma in (A) and (B). The levels of CDH1 mRNA remain unchanged across the major stages of colon (C), pancreatic (D), lung (E), and endometrial (F) carcinomas. **, p < 0.01. Supplementary Figure 2. E-cad protein is either upregulated or remains unchanged in most major tumor stages. E-cad levels are either significantly upregulated or remain unchanged in distinct stages of endometrial carcinoma (A), lung carcinoma (B), ovarian carcinoma (C), head/neck carcinoma (D), and colon carcinoma (E and F) except for stage 1 of head/neck carcinoma (D) compared to the corresponding normal tissues. Numbers of tissues used in the analysis are: endometrial carcinoma: normal, n=31, stage 1,n=74, stage 2, n=8, stage 3, n=15; lung cancer: normal, n=11, stage 1, n=59, stage 2, n=30, stage 3, n=21; ovarian cancer: normal, n=25, stage 1, n=2, stage 3, n=75, stage 4, n=16; head/neck carcinoma: normal, n=71, stage 1, n=7, stage 2, n=25, stage 3, n=75, stage 4, n=4; colon carcinoma: normal, n=100, mucinous, n=19, non-mucinous, n=77, stage 1, n=10, stage 2, n=39, stage 3, n=40, stage 4, n=8. *, p < 0.05, **, p < 0.01. [file 12885_2023_10916_MOESM1_ESM.pdf]

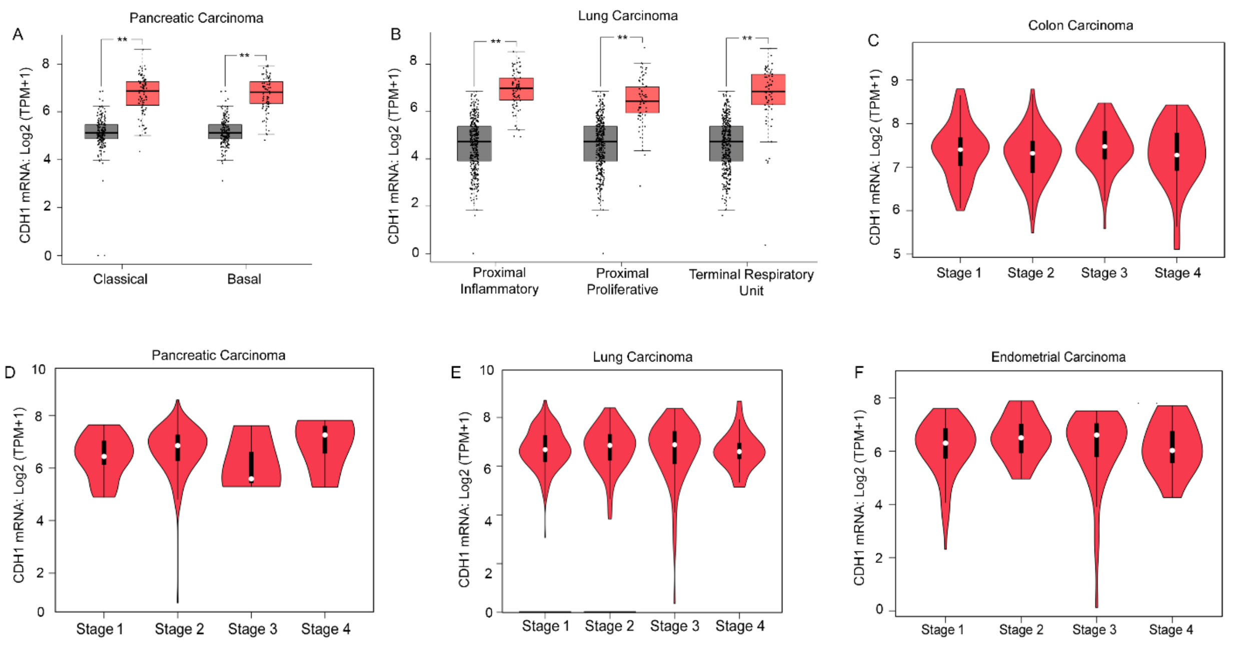

Supplementary Figure 1. *CDH1* mRNA is significantly upregulated in some carcinoma subtypes and remains unchanged across major tumor stages of most carcinomas. *CDH1* mRNA levels are significantly upregulated in the two subtypes of pancreatic carcinoma: classical (T=86) and basal (T=65) compared to the normal tissues (N=171) (A) and in the three subtypes of lung carcinoma: proximal inflammatory (T=78), proximal proliferative (T=58), and terminal respiratory unit (T=68) compared to the normal tissues (N=347) (B). Grey, normal; red, carcinoma in (A) and (B). The levels of *CDH1* mRNA remain unchanged across the major stages of colon (C), pancreatic (D), lung (E), and endometrial (F) carcinomas. **, $p < 0.01$.

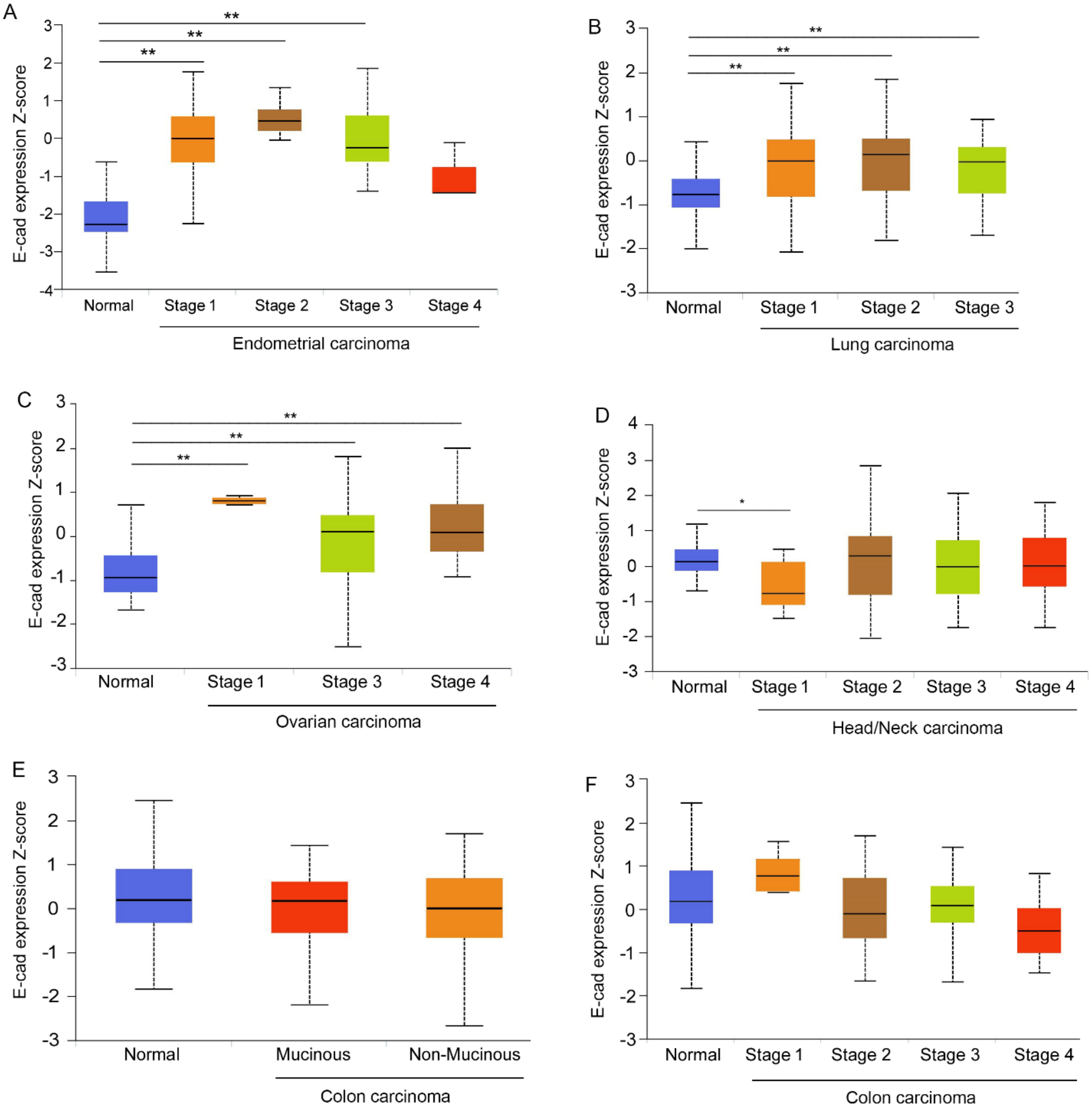

Supplementary Figure 2. E-cad protein is either upregulated or remains unchanged in most major tumor stages. E-cad levels are either significantly upregulated or remain unchanged in distinct stages of endometrial carcinoma (A), lung carcinoma (B), ovarian carcinoma (C), head/neck carcinoma (D), and colon carcinoma (E and F) except for stage 1 of head/neck carcinoma (D) compared to the corresponding normal tissues. Numbers of tissues used in the

analysis are: endometrial carcinoma: normal, n=31, stage 1,n=74, stage 2, n=8, stage 3, n=15; lung cancer: normal, n=11, stage 1, n=59, stage 2, n=30, stage 3, n=21; ovarian cancer: normal, n=25, stage 1, n=2, stage 3, n=75, stage 4, n=16; head/neck carcinoma: normal, n=71, stage 1, n=7, stage 2, n=25, stage 3, n=75, stage 4, n=4; colon carcinoma: normal, n=100, mucinous, n=19, non-mucinous, n=77, stage 1, n=10, stage 2, n=39, stage 3, n=40, stage 4, n=8. *, $p < 0.05$, **, $p < 0.01$.
